# Biofiltration of Emerging Contaminants as a Sustainable Pest Management Strategy and Its Impact on *Corbicula fluminea*

**DOI:** 10.3390/ph19060870

**Published:** 2026-05-30

**Authors:** André M. P. T. Pereira, Eva Domingues, Liliana J. G. Silva, Andreia Freitas, Paula V. Morais, Sara Domingues, Tiago Lima, Gabriela J. da Silva, Ana Paula Chung, João Gomes

**Affiliations:** 1 LAQV, REQUIMTE, Laboratory of Bromatology and Pharmacognosy, Faculty of Pharmacy, University of Coimbra, Polo III, Azinhaga de Stª Comba, 3000-548 Coimbra, Portugal; ljgsilva@ff.uc.pt; 2 CERES, Department of Chemical Engineering, Faculty of Sciences and Technology, University of Coimbra, Rua Sílvio Lima, Polo II, 3030-790 Coimbra, Portugal; evadomingues@eq.uc.pt (E.D.); jgomes@eq.uc.pt (J.G.); 3 LAQV, REQUIMTE, Rua Dom Manuel II, Apartado 55142, 4051-401 Porto, Portugal; andreia.freitas@iniav.pt; 4 INIAV, I.P., Rua dos Lágidos, Lugar da Madalena, Vairão, 4485-655 Vila do Conde, Portugal; 5 CEMMPRE, ARISE, Department of Life Sciences, University of Coimbra, Calçada Martim de Freitas, 3000-456 Coimbra, Portugal; pvmorais@ci.uc.pt (P.V.M.); ana.chung@uc.pt (A.P.C.); 6 Faculty of Pharmacy of University of Coimbra, University Coimbra, Polo III, Azinhaga de Stª Comba, 3000-458 Coimbra, Portugal; saradomingues@ff.uc.pt (S.D.); gjsilva@ci.uc.pt (G.J.d.S.); 7 CNC-UC—Center for Neuroscience and Cell Biology, University Coimbra, 3004-517 Coimbra, Portugal; 8 CIBB—Centre for Innovative Biomedicine and Biotechnology, University Coimbra, 3004-548 Coimbra, Portugal; 9 Comprehensive Health Research Center (CHRC), University of Évora, 7004-516 Évora, Portugal; tiago.lima@uevora.pt; 10 Department of Medical and Health Sciences, School of Health and Human Development, University of Évora, 7004-516 Évora, Portugal

**Keywords:** antibiotic-resistant bacteria, pharmaceuticals, microbiome, wastewater disinfection, decontamination

## Abstract

**Background/Objectives**: Water scarcity is driving the development of strategies for treating municipal wastewater (MW) to enable its safe reuse. Nonetheless, MW contains contaminants of emerging concern (CECs), such as pharmaceuticals and antimicrobial-resistant (AMR) bacteria, which require innovative treatment technologies. In this context, *Corbicula fluminea*, an invasive freshwater clam, presents a high biofiltration capacity, and its environmental impact could be mitigated by assigning it a beneficial role in wastewater treatment. **Methods**: The ability of *C. fluminea* to remove chemical and biological CECs from real MW secondary-treated effluents was assessed. The effects of real wastewater on the clams’ microbiome and on colony-forming unit (CFU) counts in their soft tissues were also assessed. **Results**: Under real conditions, the clams achieved over 73% removal for 3 chemical CECs after 24 h, with an average removal of approximately 39%. The clams showed recovery of both CFU counts and microbial community composition, dominated by opportunistic and stress-tolerant groups in the presence of pharmaceuticals. The removal of multidrug-resistant bacteria was evaluated; despite real wastewater reducing clearance rates, the clams significantly reduced these bacteria within 24 h. **Conclusions**: These results demonstrate that *C. fluminea* can serve as an effective polishing treatment, improving effluent quality, supporting control of this invasive species.

## 1. Introduction

Water scarcity is one of the major challenges of the century, particularly in regions experiencing high water stress, such as arid and semi-arid regions. This concern is related to global industrialisation and exponential population growth, which together increase the demand for high-quality water and exert significant pressure on available water sources [1]. As a result, the decline in water quality due to the intensive discharge of industrial and municipal wastewater into water sources is significant. As a result, its safe availability is compromised [2]. Therefore, enhancing wastewater reclamation strategies through the safe reuse of treated wastewater is crucial to mitigate these impacts. However, current wastewater treatment technologies, both industrial and municipal, need to be improved to meet environmental safety standards and comply with legislative requirements.

Municipal wastewater treatment plants (MWTPs) can be identified as the primary pathway of pollution for pharmaceutical and personal care products (PPCPs), chemicals belonging to the wide group of contaminants of emerging concern (CECs). One study identified and quantified several PPCPs from various MWTPs, with concentrations ranging from micrograms to nanograms per litre across different water sources [3]. Recalcitrant PPCPs present in municipal wastewater are difficult to remove by conventional methods, which mainly rely on microbiological treatment systems. Continuous discharge of these non-biodegradable and bioaccumulative compounds can promote their persistence and increase their concentrations in water sources, posing a significant risk to aquatic ecosystems [4].

The removal of PPCPs, especially antibiotics, from wastewater is particularly important due to their significant environmental impact. Antibiotics can contribute to the development of antibiotic-resistant bacteria, which poses a major public health threat. Additionally, these compounds can disrupt aquatic ecosystems by affecting the growth and reproduction of non-target organisms, such as algae and fish. For instance, antibiotic residues can alter microbial communities in water bodies, leading to reduced biodiversity and impaired ecosystem functions. Ensuring effective removal of antibiotics is crucial to mitigate these adverse effects and protect environmental and human health [5]. Antimicrobial resistance (AMR) should be minimised due to its impact on bacterial communities and human health when present in wastewater [6].

Although current legislation on wastewater reuse does not address specific contaminants, the impact of PPCPs on human and environmental health, as well as the presence of AMR, is well documented [7,8]. Therefore, it is essential to explore suitable alternatives to enhance conventional treatments for the removal of PPCPs, including antibiotics and antibiotic-resistant bacteria (biological CECs). Advanced oxidation processes have been identified as a promising technology for reducing PPCPs and enteric pathogens, but they can also incur significant application costs [1].

*Corbicula fluminea* has been identified as a significant environmental concern in Europe due to its invasive nature [9]. Consequently, removing these invasive species from the affected areas to prevent their competition with native species for food and space is essential. In this context, the biofiltration capacity of *C. fluminea* could serve as a valuable tool for removing emerging contaminants from wastewater. These clams can withstand adverse conditions, making them an interesting solution for municipal wastewater treatment. Other authors demonstrated this remarkable capacity of *C. fluminea* while effectively removing compounds from olive mill wastewater [10]. Similarly, studies have also proved that it can be effectively applied to remove organic content from swine wastewater [11].

In this way, the application of biofilter species such as *C. fluminea* may be a suitable alternative for improving municipal wastewater treatment. The use of this invasive species for the removal of emerging contaminants could be considered a pest management approach, as individuals could be removed from invaded sites. Nonetheless, its biofiltration performance can be affected by various wastewater constituents, including organic and inorganic compounds such as PPCPs, microorganisms, metals, and organic matter [3]. Moreover, the clam’s microbiome may support the metabolization of some contaminants or organic compounds present in wastewater. Therefore, the impact of wastewater composition on the clam’s microbiome should be assessed.

Currently, studies investigating the use of *C. fluminea* for the removal of PPCPs and antibiotic-resistant bacteria in real municipal wastewater remain scarce. The objectives of this study were to identify the PPCPs present in municipal wastewater and evaluate their removal efficiency, along with that of a multidrug-resistant *Escherichia coli* strain, using clam biofiltration. Additionally, this study aimed to assess the impact of PPCPs and other wastewater constituents on the clam microbiome.

## 2. Results and Discussion

### 2.1. Pharmaceuticals Biofiltration

Of the 19 pharmaceuticals found in the wastewater effluent used, belonging to five therapeutic groups, 13 were removed (average of 39.5% and total average of 36.5%) by the clams (Figure S1, Supplementary Information). Only six pharmaceuticals were not removed (losartan, cefapirin, three sulfonamides, and carbamazepine).

**Figure 1:**
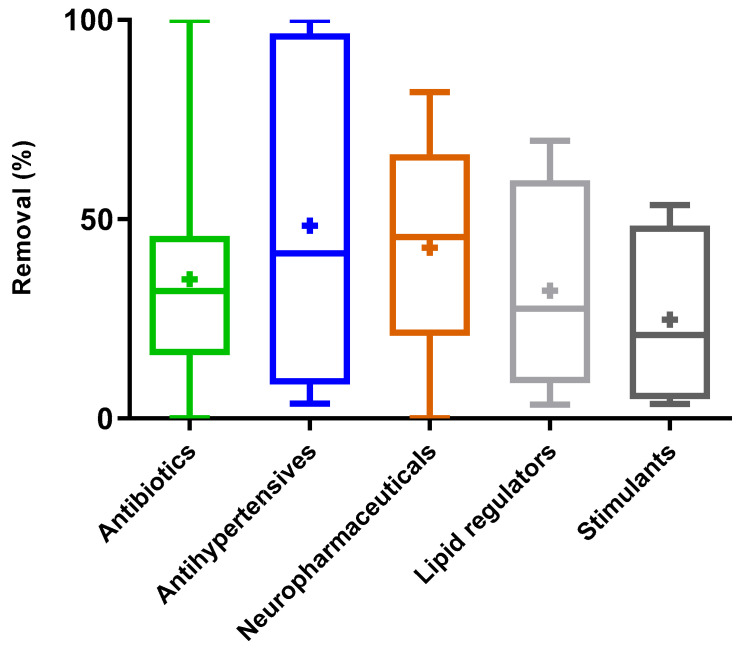
Boxplot showing clam removal efficiency, calculated by comparison with the blank, for each therapeutic group across all assays. Whiskers represent the minimum and maximum values, boxes indicate the first quartile, median, and third quartile, and the “+” symbol indicates the mean.

As shown in Figure 1, when considering the different therapeutic groups, the highest average removal efficiency was observed for antihypertensives (48.3%), followed by neuropharmaceuticals (42.9%) and antibiotics (35.0%). Stimulants showed the lowest removal efficiency (24.8%). However, only one drug from this group was tested, so other stimulants may exhibit different removal rates. No statistically significant differences were observed among the therapeutic groups. This suggests that, although physicochemical characteristics vary, even within the same therapeutic group, this treatment has the potential to remove a wide range of pharmaceuticals.

The drugs with the highest removal rates were, in descending order, propranolol (96.5%), citalopram (76.9%), and ciprofloxacin (74.0%) (Figure S1, Supplementary Information). The least efficiently removed drugs were alpha-hydroxyalprazolam (3.8%), irbesartan (7.7%), and azithromycin (11.3%). The remaining drugs showed removal rates ranging from 20.0 to 65.0%. Statistically significant differences were observed only between propranolol and irbesartan (*p* = 0.0134), and between propranolol and alpha-hydroxyalprazolam (*p* = 0.0092).

To contextualize the proposed methodology, MWTP performance in pharmaceutical removal was used as a qualitative benchmark and categorized into different levels according to the specificity and quality of the implemented methods. The removal efficiency of a conventional MWTP is around 64%, while an advanced MWTP has an average removal efficiency of 90%, and in some cases, it can reach up to 100% [12]. The obtained removal percentages of some therapeutic groups, particularly neuropharmaceuticals, are closer to the range reported for MWTP, although the MWTP removal rates result from the combined effects of multiple treatment processes within the plant. The other values are somewhat lower but still close to the reported MWTP removal ranges, except for antibiotics and stimulants. When comparing clam-based treatment with conventional MWTPs, carbamazepine stands out as a molecule that is highly resistant to different removal treatments [12,13].

As expected, the average values from the 24 h hydraulic retention time trials (37.3%) were lower than those from the 48 h trials (41.7%) (Figure S2, Supplementary Information). However, there was no significant difference between these two sets of data (*p* = 0.5671). This is advantageous in terms of cost and efficiency, as 24 h hydraulic retention treatment enables shorter treatment times, smaller equipment, and lower energy consumption, facilitating its application in low-income countries. Although this removal efficiency may seem low compared with that of an MWTP, this percentage reflects the outcome of a single method applied over a short hydraulic retention time to MWTP effluent. Therefore, improved performance is expected to be achievable with further optimization [12].

Regarding the assays comparing new and reused clams, the removal percentages from both trials were similar. Moreover, the average removal percentage achieved with reused clams (40.7%) surpassed that observed in trials with new clams (38.3%) (Figure S3, Supplementary Information). Therefore, clam reuse appears to be feasible, as the removal rates did not differ significantly from those observed with clams in their initial use (*p* = 0.3582). Furthermore, reuse prevents the formation of by-products and simplifies system implementation.

These results confirm the biofiltering capacity of clams, with significant pharmaceutical removal rates observed in MWTP effluent samples. This could support the production of reclaimed wastewater effluents suitable for reuse in street cleaning, agriculture, municipal gardens, golf courses, irrigation, etc.

Other authors have explored the possibility of using microalgae to treat pharmaceuticals in MWTP effluents in a study similar to the present one. The average removal efficiency ranged from 64.0 to 75.3%, with a hydraulic retention time of 24 h. The therapeutic groups with the lowest removal rates were antibiotics and antihypertensives, with ciprofloxacin removal (53.2%) being lower than that observed in the clam treatment evaluated in this study (74.0%). The microalgae treatment was conducted in an actual MWTP, with controlled experimental conditions. Although algae-based systems have shown potential for contaminant removal, their performance is often less reproducible than that of bivalve-based systems. This reduced reproducibility is mainly due to the high variability among algal cultures: even adjacent tanks may contain algal populations with distinct species composition, growth rates, or physiological states. Moreover, algal experiments typically require small, transparent tanks to ensure adequate light penetration, which further limits the scalability and uniformity of the results [14]. In contrast, bivalve-based treatments tend to provide more consistent and comparable outcomes under similar experimental conditions [15].

Compared with other nature-based solutions, constructed wetlands have shown average pharmaceutical removal efficiencies between 21% and 93%. Their performance depends on compound properties and removal mechanisms, including biodegradation, sorption, plant uptake and photodegradation. In this context, the present *C. fluminea* treatment may represent a more compact biological approach, achieving relevant removal within a 24 h exposure period [16].

Advanced oxidation processes may achieve high pharmaceutical removal within much shorter treatment times than biological systems. Light-driven advanced oxidation processes (AOPs) applied to real urban wastewater removed carbamazepine, ibuprofen and ciprofloxacin with an average removal of approximately 90 to 100% after 40 min of treatment. Individually, removals ranged from 80.4 to 100% for carbamazepine, 89.83 to 100% for ibuprofen, with complete removal for ciprofloxacin. However, these processes require higher energy inputs and chemical addition than the 24 h *C. fluminea* treatment [17].

Another study, similar to the current one, tested the capacity of the bivalve *Dreissena polymorpha* to remove pharmaceuticals and illicit drugs from wastewater effluents. Removal efficiencies ranged from −3.4% to 33.3% [18]. The overall average removal in that study was 9%, which is significantly lower than that observed in the present study (36.6%). However, the trials with *D. polymorpha* had an exposure time of only 6 h [18], compared with the 24 h exposure used in the *C. fluminea* trials.

Some uncertainties remain regarding the maintenance requirements and efficiency of implementing this treatment in an MWTP, particularly concerning the disposal of the clams’ depuration water and the prevention of clams escaping their cages, which could contaminate the aquatic environment. Regarding depuration water, one solution could be to reintroduce it into the MWTP for treatment. As for preventing the clams from escaping the plant, a range of solutions is available, some as simple as installing a mesh network, narrow enough to allow treated water to pass while retaining the various life stages of *C. fluminea*. There is also the possibility of converting clams that can no longer remove pharmaceuticals into biomass, which could provide additional revenue for the treatment plant.

### 2.2. Microbiome Impact

#### 2.2.1. Impact of Effluent Exposure on Culturable Bacteria from *C. fluminea* Soft Tissue

**Figure 2:**
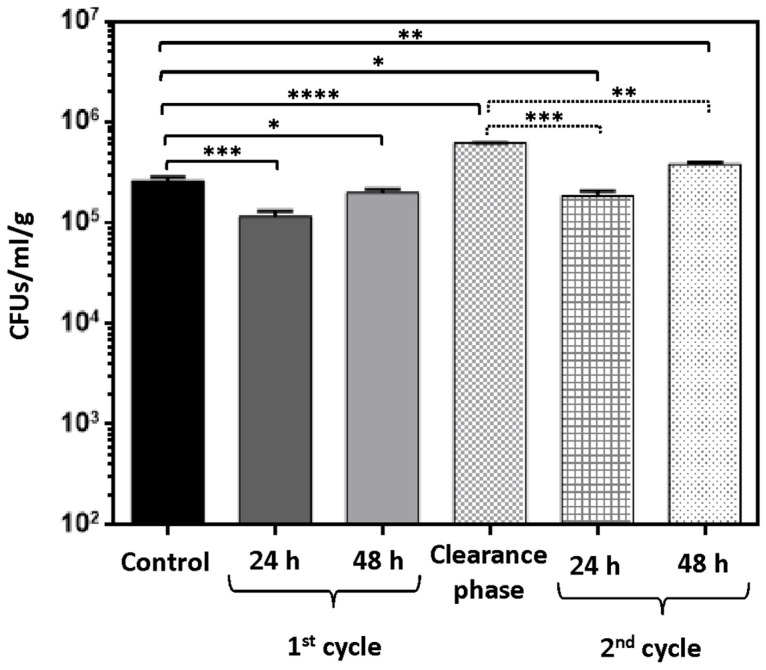
Colony-forming unit (CFU) counts in the soft tissues of *C. fluminea* across two wastewater exposure cycles. Clams were exposed for 24 h and 48 h in each cycle, with a 7-day depuration period between cycles. The control group consisted of non-exposed clams maintained in dechlorinated water. Asterisks indicate statistically significant differences relative to the control group in the first cycle and to the depuration phase in the second cycle (* *p* < 0.05; ** *p* < 0.01; *** *p* < 0.001; **** *p* < 0.0001).

Exposure of *C. fluminea* to effluent containing a mixture of pharmaceuticals reduced the bacterial load in clam soft tissue, as shown by colony-forming unit (CFU) counts on R2A medium (Figure 2). In the first exposure cycle, after 24 h of exposure, CFU counts decreased to 1.2 × 10^5^ compared with 2.6 × 10^5^ in control clams. At 48 h, CFU counts increased slightly to 2.0 × 10^5^ but remained below control levels. These results suggest an initial suppressive effect of the effluent on the clam-associated bacterial community [19,20,21].

After a 7-day clearance period in dechlorinated water, CFU counts increased significantly to 6.1 × 10^5^, more than double the control CFU counts (Figure 2). This recovery indicates that, in the absence of effluent stress, opportunistic or effluent-resistant strains that survived the initial exposure were able to proliferate, likely due to reduced microbial competition [22,23].

In the second exposure cycle, the CFU counts decreased again to 1.9 × 10^5^ at 24 h, followed by an increase to 3.8 × 10^5^ at 48 h (Figure 2). This pattern, similar to the first cycle, reflects the dynamic interaction between the environmental contaminants and clam-associated bacteria. It also highlights the ability of *C. fluminea* to maintain a functional response (e.g., microbial load suppression during exposure and recovery afterward), supporting its suitability for use in repeated exposure experiments [24].

#### 2.2.2. Effect of Effluent Exposure on Bacterial Composition and Diversity of *C. fluminea* Soft Tissue

**Figure 3:**
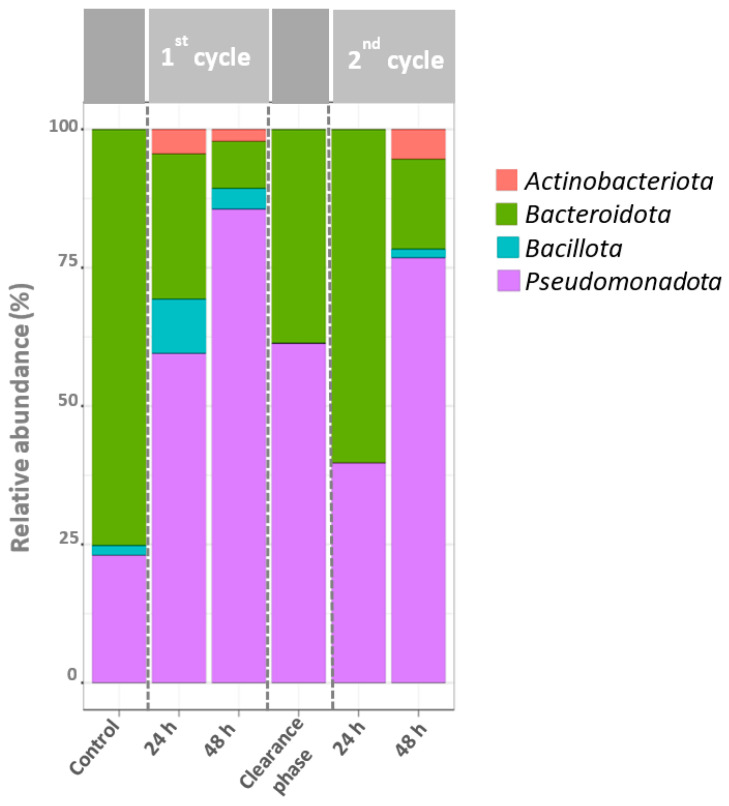
Relative abundance of bacterial phyla in the soft tissues of *Corbicula fluminea* after 24 h and 48 h of wastewater exposure across two exposure cycles. Non-exposed clams served as controls, and a depuration phase was included between cycles.

Exposure of *C. fluminea* to pharmaceutical effluent induced pronounced shifts in the bacterial community structure of the clam soft tissue, with marked differences observed between exposure cycles and across time points. At the phylum level, the microbiome was dominated by *Pseudomonadota* (23.1–85.6%) and *Bacteroidota* (8.5–75.2%), while *Bacillota* and *Actinobacteriota* were present at lower relative abundances, not exceeding 10% (Figure 3).

**Figure 4:**
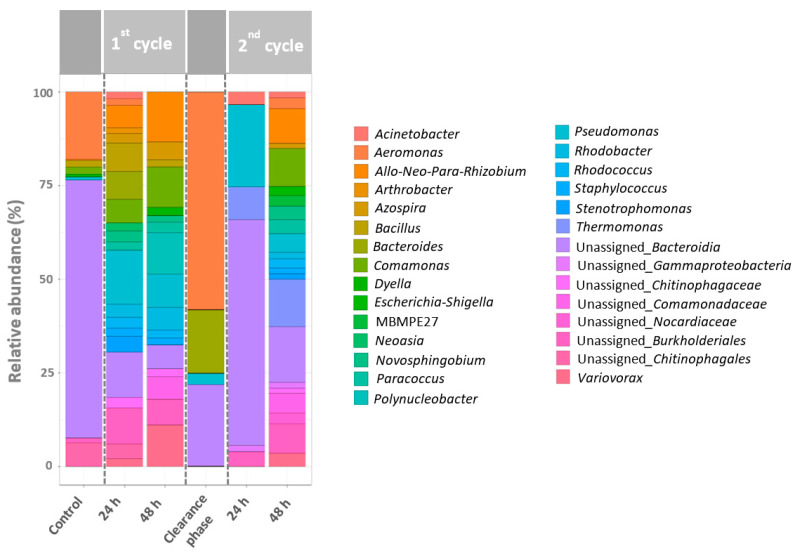
Relative abundance of bacterial genera in soft tissues of *Corbicula fluminea* after two cycles of exposure to effluent containing a mixture of pharmaceuticals. In each cycle, clams were exposed for 24 h and 48 h, while non-exposed clams served as controls. A 7-day clearance phase in dechlorinated water was included between the two exposure cycles. Only genera with relative abundances higher than 1.0% are shown.

The bacterial community of unexposed clams was dominated at the genus level by *Unassigned_Bacteroidia* (68.9%), with moderate abundances of *Aeromonas* (18%) and *Unassigned_Cytophagales* (6.3%) (Figure 4). The high baseline abundance of members of the class *Bacteroidia* is consistent with previous studies [25,26] and aligns with their known ability in organic matter degradation, which enhances nutrient availability and microbiome stability [27,28].

Effluent exposure exerted strong selective pressure, causing a marked decrease in *Unassigned_Bacteroidia* to 12.0% (24 h) and 6.4% (48 h) during the first cycle (Figure 4). However, its abundance increased to 21.8% after the 7-day clearance phase, demonstrating significant resilience once the pharmaceutical stressor was removed [28,29]. In the second exposure cycle, *Unassigned_Bacteroidia* reached 60.2% at 24 h but decreased to 14.9% by 48 h (Figure 4), suggesting that prolonged exposure compromised its stability and persistence, consistent with findings showing that extended stress can destabilize microbial communities [19,20].

*Aeromonas* also showed dynamic shifts in abundance. During the first exposure, their abundance decreased drastically from 18.0% (control) to 1.8% at 24 h and was undetectable at 48 h, probably due to the combined effects of pharmaceutical toxicity and competitive displacement by more resistant taxa [30]. Despite the metabolic flexibility of *Aeromonas* species, enabling adaptation to diverse environmental stresses [31,32], the complex mixture of pharmaceuticals likely disrupted their metabolic pathways, reducing growth and survival [30,33]. After the clearance phase, *Aeromonas* became dominant, comprising 58.1% of the community (Figure 4). This recovery can be attributed to the opportunistic growth of resistant strains and the establishment of biofilms, facilitating recolonization [34,35]. However, during the second exposure, the genus *Aeromonas* was absent at 24 h or represented only 2.8% at 48 h, indicating cumulative detrimental effects from repeated pharmaceutical stress [30,36].

*Pseudomonas* and *Variovorax* showed strong growth after exposure, acting as early opportunists. *Pseudomonas* was enriched at 24 h in both cycles (14.4 and 21.9%, respectively), while *Variovorax* became more abundant at 48 h in the first cycle (11.1%). Their relative abundance declined at later stages, suggesting a strategy of rapid exploitation of pharmaceuticals as substrates, but limited persistence under sustained stress. This pattern is consistent with the well-documented ability of *Pseudomonas* to degrade complex compounds and tolerate chemical stressors [37,38], and the metabolic versatility of *Variovorax* in degrading xenobiotics, including pharmaceuticals [39].

In contrast, the relative abundance of *Allorhizobium-Neorhizobium-Pararhizobium-Rhizobium* group, *Comamonas, Unassigned_Comamonadaceae,* and *Unassigned_Burkholderiales* increased (5.0–13.0%) at 48 h in both exposure cycles (Figure 4). These taxa are recognized for their metabolic versatility and capacity to degrade a wide spectrum of xenobiotics [40,41,42,43], appear to act as late degraders, potentially targeting more recalcitrant pharmaceutical compounds or breakdown products once early substrates have been consumed.

The presence of *Thermomonas* only in the second exposure cycle, where it reached 12.6% at 48 h, further indicates that repeated exposure promotes selection for resilient degraders capable of using diverse pharmaceutical residues [44,45].

Alpha diversity metrics were evaluated to assess how microbial community diversity responded to the two effluent exposure cycles (Table 1). The control sample exhibited the highest observed richness (194) and Shannon index (4.51), reflecting a taxonomically rich community, although evenness was moderate (0.47), due to dominance by *Unassigned_C_Bacteroidia* and *Aeromonas*.

During the first exposure cycle, observed richness was strongly reduced to 61 amplicon sequence variants (ASVs) at 24 h and further to 25 ASVs at 48 h. Despite this reduction, evenness increased (0.79–0.78), suggesting that the effluent imposed strong selective pressure favoring opportunistic and stress-tolerant groups such as *Pseudomonas*, *Comamonas* and *Unassigned_Burkholderiales*, which were more evenly distributed. During the clearance phase, richness recovered to 126 ASVs, but the Shannon index decreased (2.26), and evenness declined drastically (0.08) due to the dominance of *Aeromonas*, *Bacteroides, and Unassigned_Bacteroidia.* In the second exposure cycle, richness reached the lowest value, 14 ASVs at 24 h, but increased to 78 ASVs. At the same time, Shannon index increased to 4.13, comparable to the control, and evenness reached its highest value (0.80), indicating a more balanced and heterogeneous community.

Overall, the partial recovery of microbial diversity during repeated exposure suggests adaptive microbiome restructuring under pharmaceutical stress. Repeated exposure also enriched bacterial taxa associated with xenobiotic degradation and chemical stress tolerance, which could potentially support host-associated microbial functions and long-term filtration performance. However, because functional and physiological parameters were not directly assessed, these interpretations remain speculative and require further long-term investigation.

### 2.3. Biological CEC Biofiltration

**Figure 5:**
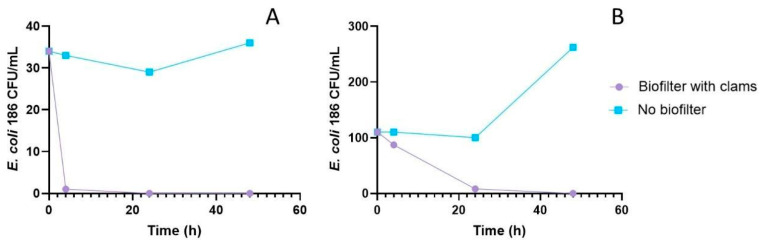
Biofiltration of multidrug-resistant *E. coli* 186 by *Corbicula fluminea* over time: (**A**) in synthetic effluent; (**B**) in real municipal effluent.

The evaluation of biofiltration capacity against the antibiotic-resistant bacteria was first determined under synthetic conditions and later using real secondary wastewater. In the first case, the dechlorinated water was spiked with 10^5^ CFU/mL of multidrug-resistant (MDR) *E. coli* 186 and submitted to biofiltration. An abrupt reduction in the MDR *E. coli* 186 was already seen after 4 h of biofiltration, while the number of bacteria remained stable in the absence of clams (Figure 5A). This test allowed the determination of the clearance rate of biofiltration of *C. fluminea* when in contact with such bacteria, which was 11 mL/hour per clam. A previous study with antibiotic-susceptible *E. coli* ATCC 25922 has achieved higher clearance rates [46], suggesting that resistance patterns may influence the biofiltration capacity of *C. fluminea*.

When added to real municipal effluent, a substantial decrease in the number of MDR *E. coli* 186 colonies (93%) was observed after 24 h (Figure 5B). *D. polymorpha*, known as the zebra mussel, has also been shown to efficiently remove endogenous *E. coli* from municipal wastewater [47]. As expected, due to the higher contamination of the effluent, the clearance rate was lower than in the synthetic effluent and corresponded to 1.36 mL/hour per clam at 24 h. In fact, the presence of organic matter when real wastewater is used can reduce the effectiveness of clam biofiltration [11,48]. This can be related to the presence of suspended particles and even organic matter, which can inhibit the biofiltration activity [49,50].

Complete removal of MDR *E. coli* was observed after 48 h of contact between the effluent and clams, while the number of bacteria remained high in the absence of the biofilters.

Overall, the biofiltration with *C. fluminea* for 24 h already showed an efficient removal rate of pharmaceuticals and antibiotic-resistant bacteria, which did not significantly increase at 48 h. As filter-feeding bivalves, clams can accumulate microorganisms that are present in the water, including antibiotic-resistant bacteria, and act as environmental reservoirs of resistance. Therefore, the use of clams as biofilters needs to be coordinated with appropriate management to prevent their release back into the environment.

## 3. Materials and Methods

### 3.1. Biofiltration Experiments

*C. fluminea* clams used in the experiments were collected from a freshwater canal located in the central region of Portugal (40°25′06.90″ N, 8°44′13.18″ W). To collect the clams, sediment from the canal bed was sieved through a mesh bag to effectively separate the bivalves from the surrounding substrate. After collection, the clams were thoroughly rinsed with dechlorinated tap water to remove debris adhering to their shells. The specimens were then acclimated under controlled laboratory conditions for two weeks. During this depuration period, the clams were maintained at a constant temperature of 20 ± 2 °C under a regulated photoperiod of 16 h light and 8 h dark, with continuous aeration to ensure adequate dissolved oxygen levels. The culture water was renewed twice weekly using dechlorinated water to facilitate natural purification processes and remove residual contaminants. Following acclimation, the clams were used in biofiltration experiments. Individuals were introduced into beakers containing real wastewater and maintained under the same environmental conditions (20 ± 2 °C, 16 h light:8 h dark photoperiod, and continuous aeration). The experiments were conducted using a standardized density of 80 clams per litre of wastewater (1:80). To ensure consistency, only clams with shell lengths ranging from 16 to 22 mm were selected for the trials. These parameters were analysed in a previous work [23].

Control experiments for each condition were performed using the same wastewater under identical conditions but without clams to evaluate removal efficiency. To assess the baseline health and natural mortality rate of the clams, control assays were conducted using dechlorinated tap water instead of wastewater, under identical conditions. These controls facilitated accurate evaluation of clam mortality that could be attributed specifically to wastewater exposure rather than to environmental stressors.

### 3.2. Chemical CEC Quantification

The analytical methodology applied for the determination of 63 pharmaceutical compounds, belonging to eight therapeutic categories (analgesics, antibiotics, neuropharmaceuticals, antidepressants, antihypertensives, stimulants, lipid-modifying agents and anti-inflammatory drugs), followed procedures previously described in the literature (Table S1, Supplementary Information) [15,51]. The method had been fully validated beforehand, presenting limits of detection between 0.01 and 3.47 ng/L.

Immediately after sampling, water samples were stored at −20 °C until analysis. Prior to extraction, samples were thawed, acidified to pH 2 using formic acid, and sequentially filtered through 0.45 µm and 0.22 µm pore size membrane filters (Filter-Lab, Johannesburg, South Africa). For the extraction step, 500 mL of each filtered sample was spiked with 500 µL of an internal standard solution containing sulfameter (10 µg/mL, purity > 99%, Sigma-Aldrich, Saint Louis, MO, USA).

Sample clean-up and preconcentration were carried out by solid-phase extraction using Oasis HLB cartridges (200 mg, 6 mL; Waters, Milford, MA, USA). After sample percolation, the cartridges were rinsed with 5 mL of a methanol:water solution (10:90, *v*/*v*) and subsequently dried under low vacuum. Elution was performed with methanol (≥99.9%, Honeywell, Charlotte, NC, USA), and the resulting extracts were evaporated to dryness under a gentle nitrogen stream at 40 °C. The residues were reconstituted in 500 µL of aqueous formic acid (0.1%, *v*/*v*; mobile phase A; Merck, Darmstadt, Germany) and filtered using PVDF Mini-UniPrep™ filters (0.45 µm) (Whatman, Marlborough, MA, USA).

Chromatographic analysis was conducted by injecting 10 µL of the final extract into a UHPLC-ToF-MS system composed of a Nexera X2 UHPLC (Shimadzu, Kyoto, Japan) coupled to a TripleTOF™ 5600+ high-resolution mass spectrometer (Sciex, Framingham, MA, USA). Separation was achieved using a reversed-phase Acquity UPLC HSS T3 column (1.8 µm, 2.1 × 100 mm; Waters, Milford, MA, USA), maintained at 40 °C. The mobile phases consisted of (A) 0.1% formic acid in water and (B) acetonitrile, delivered at a flow rate of 500 µL/min. The gradient program started at 97% A, decreasing to 40% A over the first 5 min, followed by a reduction to 0% A between 5 and 9 min. The initial conditions were restored between 9 and 10 min and maintained until the end of the 12 min run (Table S2, Supplementary Information).

Mass spectrometric detection was performed using electrospray ionization in positive mode (ESI+), operating in full-scan acquisition over a mass range of 100–920 Da. Data acquisition was carried out using Analyst^®^ TF 1.7 software, while data processing and compound identification were performed with PeakView™, LibraryView™, and MultiQuant™ software packages (SCIEX, Framingham, MA, USA). Compound identification was based on accurate mass measurements (mass error ≤ 5 ppm), retention time deviation below 1% relative to reference standards (Commission Regulation (EU) 2021/808) [52], and isotope pattern matching, allowing a maximum deviation of 10% between experimental and theoretical distributions.

### 3.3. Microbiome Assessment

#### 3.3.1. Colony-Forming Unit (CFU) Counts and Microbiome Assessment of *C. fluminea* Soft Tissue Exposed to Real Wastewater

*C. fluminea* clams were exposed to real wastewater from the secondary stage of treatment for 24 h and 48 h periods. Following this initial exposure cycle, the clams underwent a 7-day clearance phase in dechlorinated water. After the clearance period, they were subjected to a second cycle of wastewater exposure for the same periods as in the first cycle. The control group consisted of *C. fluminea* maintained in dechlorinated water for depuration after collection, without exposure to the wastewater.

Clam shells were surface-sterilized with 70% ethanol, and soft tissues were aseptically removed using flame-sterilized tweezers. Soft tissues from three clams were pooled into a composite sample, cut into small fragments, and mechanically disrupted with a scalpel. This sample was homogenized in a shaking incubator at 1200 rpm for 30 min, followed by ultrasonic treatment in a bath sonicator operating at 45 kHz and 80 W for 3 min to enhance bacterial release. Serial 10-fold dilutions of the tissue homogenate were prepared in sterile saline, and 100 μL from each dilution was plated in triplicate onto R2A agar. After incubation at 25 °C for 24 h, colony-forming units (CFUs) were enumerated, averaged across triplicates, and normalized to the pooled soft tissue weight, with results expressed as CFU/mL/g.

#### 3.3.2. Microbiome Assessment Through 16S rRNA Amplicon Sequencing and Bioinformatic Analysis

Genomic DNA was isolated using the E.Z.N.A.^®^ Bacterial DNA Kit (Omega Bio-Tek, Norcross, GA, USA), following the protocol provided by the manufacturer. Amplicon libraries were prepared using a two-step PCR workflow designed to amplify the V3–V4 hypervariable region of the 16S rRNA gene. Sequencing was carried out on an Illumina MiSeq system (MiSeq Control Software v4.0/RTA v1.18.54) employing the MiSeq v3 reagent kit with paired-end reads (2 × 300 bp), at the University of Copenhagen, Denmark.

Bioinformatic processing of raw sequencing data was performed using QIIME 2 version 2024.10 [53]. Initial processing included demultiplexing and quality control using the q2-demux plugin, followed by sequence denoising and amplicon sequence variant (ASV) inference using DADA2 [54]. To improve taxonomic resolution, a custom feature classifier was trained on the SILVA 138 reference sequence and taxonomy databases and applied through the q2-feature-classifier plugin [55,56]. Taxonomic assignment was subsequently performed at the ASV level using this trained classifier.

Downstream analyses and graphical visualizations were conducted in R version 4.4.2 using RStudio (version 2024.12.0-467). The qiime2R package [57] was employed to import and handle QIIME 2 output files within the R environment.

ASV and taxonomic data were managed with the phyloseq package [58], and data tidying and visualization were performed using the tidyverse package [59]. To minimize low-abundance noise, ASVs representing ≤1% relative abundance were excluded. The filtered dataset was then split into two subsets, and relative abundance plots were generated at the phylum and genus levels for each subset.

Alpha diversity was quantified using the Observed richness, Shannon diversity index (H′), Inverse Simpson index (1/D), and evenness (e^H/S), all computed with the estimate_richness function available in the phyloseq package.

### 3.4. Biological CEC Quantification

The biofiltration experiments were performed with the MDR strain *Escherichia coli* 186, which carries resistance to different classes of beta-lactams, colistin, and tetracyclines [60]. The strain grows in CHROMagar™ ESBL (CHROMagar, Paris, France), producing dark pink to reddish colonies.

Quantification of bacteria in water samples was done as previously described [46], with a few adaptations. Briefly, *E. coli* 186 was added to biofilters with 1000 mL of dechlorinated water or real municipal effluent and 80 clams, resulting in a final concentration of 1 × 10^5^ CFU/mL. *E. coli* 186 viability during the assay was controlled in a biofilter with 1000 mL of dechlorinated water or real municipal effluent and without clams; the effluent was also checked for the presence of strains growing on CHROMagar™ ESBL. The biofiltration evaluation was performed by removing water samples at time 0, 4 h, 24 h and 48 h, followed by filtration through a 0.2 µm mixed cellulose ester membrane (Advantec, Tokyo, Japan); when needed, the samples collected were serially diluted to achieve plate counts within the 30–300 CFU range. The filters were placed on CHROMagar™ ESBL plates and incubated at 37 °C for 24 h in the dark. Dark pink to reddish colonies were counted and the result reported as CFU/mL, where each assay was performed in duplicate. The clearance rate of each clam was calculated as previously described [46].

### 3.5. Statistical Analyses

All statistical evaluations related to pharmaceutical removal were carried out using GraphPad Prism software (version 8.4.3; GraphPad Software, Inc., San Diego, CA, USA). Data distribution was examined using the D’Agostino–Pearson normality test. As most variables failed to meet the assumptions of normality and homogeneity of variances, nonparametric statistical methods were adopted. Differences among three or more datasets were evaluated using the Kruskal–Wallis test, followed by Dunn’s post hoc multiple comparison test. Comparisons between two datasets were performed using the Mann–Whitney U test. Pharmaceutical compounds not detected in the wastewater effluent analyzed in this study were omitted from the statistical analysis.

For bacterial load measurements in clam soft tissues (CFU mL^−1^ g^−1^), differences among sampling groups were assessed using one-way analysis of variance (ANOVA). When significant effects were observed, Dunnett’s multiple comparisons test was applied to identify differences between each treatment and the control group, as well as between the clearance phase and the 24 h and 48 h time points of the second experimental cycle. In all analyses, statistical significance was defined as *p* < 0.05.

The degree of removal was determined by comparing the concentrations measured in the experimental assays with those obtained in the corresponding controls (blanks), which were conducted under identical conditions but in the absence of clams. Additionally, two complementary approaches were used. First, average removal was obtained by averaging the individual removal percentages calculated for each occurrence of each compound. Second, total average removal was expressed as the percentage difference in pharmaceutical load between the system inlet and outlet, where load was defined as the cumulative sum of all detected occurrences of each pharmaceutical at the respective sampling points.

## 4. Conclusions

The current study demonstrated that the invasive bivalve *C. fluminea* can be effectively applied as a wastewater polishing treatment, as it can remove contaminants of emerging concern within a 24 h hydraulic retention time. The results obtained after a 24 h hydraulic retention time did not differ significantly from those observed in the 48 h trials. The reuse of the clams is feasible, as the trials conducted with reused clams demonstrated removal rates comparable to those of the clams in their initial state. The removal values obtained for pharmaceuticals (39%) are approximately half of those achieved by MWTPs. These systems employ multiple treatment processes, whereas only a single method was evaluated in this study. Some therapeutic groups were less prone to removal by the clams, coinciding with groups that are more efficiently removed by conventional MWTPs. However, some pharmaceuticals, such as carbamazepine, still pose significant removal challenges in both scenarios. Moreover, this application can be seen as a pest management approach since these clams were removed from invaded sites.

The microbiome associated with *C. fluminea* exhibits a robust functional response to pharmaceutical stress, characterized by a dynamic microbial succession with initial suppression of sensitive taxa. Furthermore, the *C. fluminea* microbiome has functional plasticity, which allows the use of this species in repeated biofiltration cycles. Biofiltration with *C. fluminea* demonstrated significant removal of multidrug-resistant *E. coli* in both synthetic and real wastewater, achieving up to a 93% reduction within 24 h and complete elimination after 48 h, though clearance rates were influenced by resistance patterns and the presence of organic matter. At the same time, this approach redirects a species that otherwise harms other aquatic organisms.

This type of treatment contributes to the One Health perspective as it improves human, animal, and environmental health.

## Figures and Tables

**Table 1:** Alpha diversity index of the soft tissue microbiome of *C. fluminea* exposed to an effluent containing a mixture of pharmaceuticals.

Alpha Diversity Index	Control	Effluent Exposure 1st Cycle		Clearance Phase	Effluent Exposure 2nd Cycle	
		24 h	48 h		24 h	48 h
Observed richness (ASV)	194.0	61.0	25.0	126.0	14.0	78.0
Shannon (H’)	4.51	3.87	2.97	2.26	2.36	4.13
InvSimpson (1/D)	1.02	1.03	1.07	1.17	1.13	1.02
Evenness (e^H/S)	0.47	0.79	0.78	0.08	0.76	0.80

## Data Availability

The original contributions presented in this study are included in the article/Supplementary Material. Further inquiries can be directed to the corresponding author(s).

## Supplementary Materials

The following supporting information can be downloaded at: https://www.mdpi.com/article/10.3390/ph19060870/s1, Figure S1: Average removal for each pharmaceutical; Figure S2: Boxplot showing clam removal efficiency, calculated by comparison with the blank, for each hydraulic retention time. Whiskers represent the minimum and maximum values, boxes indicate the first quartile, median, and third quartile, and the “+” symbol indicates the mean; Figure S3: Boxplot showing clam removal efficiency, calculated by comparison with the blank, for new and reused clams. Whiskers represent the minimum and maximum values, boxes indicate the first quartile, median, and third quartile, and the “+” symbol indicates the mean; Table S1: Pharmaceutical compounds included in the scope of the method, mass detection parameters, and limits of detection (LOD) for each compound in water; Table S2: Mobile phase gradient used during chromatographic analysis.

## Abbreviations

The following abbreviations are used in this manuscript:

MW	Municipal wastewater
CECs	Contaminants of emerging concern
AMR	Antimicrobial-resistant
CFU	Colony-forming unit
MWTPs	Municipal wastewater treatment plants
PPCPs	Pharmaceutical and personal care products
ASVs	Amplicon sequence variants
MDR	Multidrug-resistant
