# Supplementary material for: Biofiltration of Emerging Contaminants as a Sustainable Pest Management Strategy and Its Impact on Corbicula fluminea
Source: Pharmaceuticals (Basel). 2026 May 30;19(6):870. doi: 10.3390/ph19060870 (PMC13306035; doi:10.3390/ph19060870)
Supplement: Supplementary file 1 [file pharmaceuticals-19-00870-s001.zip › pharmaceuticals-4302702-supplementary.pdf]

Supplementary Information

Biofiltration of Emerging Contaminants as a Sustainable Pest Management Strategy and Its Impact on *Corbicula fluminea*

André M. P. T. Pereira [1,*], Eva Domingues [2], Liliana J. G. Silva [1], Andreia Freitas [3,4], Paula V. Morais [5], Sara Domingues [6,7,8], Tiago Lima [9,10], Gabriela J. da Silva [6,7,8], Ana Paula Chung [5] and João Gomes [2]

[1] LAQV, REQUIMTE, Laboratory of Bromatology and Pharmacognosy, Faculty of Pharmacy, University of Coimbra, Polo III, Azinhaga de Stª Comba, 3000-548 Coimbra, Portugal; ljgsilva@ff.uc.pt

[2] CERES, Department of Chemical Engineering, Faculty of Sciences and Technology, University of Coimbra, Rua Sílvio Lima, Polo II, 3030-790 Coimbra, Portugal; vadomingues@eq.uc.pt (E.D.); jgomes@eq.uc.pt (J.G.)

[3] LAQV, REQUIMTE, Rua Dom Manuel II, Apartado 55142, 4051-401 Porto, Portugal; andreia.freitas@iniav.pt

[4] INIAV, I.P., Rua dos Lágidos, Lugar da Madalena, Vairão, 4485-655 Vila do Conde, Portugal

[5] CEMMPRE, ARISE, Department of Life Sciences, University of Coimbra, Calçada Martim de Freitas, 3000-456 Coimbra, Portugal; pvmorais@ci.uc.pt (P.V.M.); ana.chung@uc.pt (A.P.C.)

[6] Faculty of Pharmacy of University of Coimbra, University Coimbra, Polo III, Azinhaga de Stª Comba, 3000-458 Coimbra, Portugal; saradomingues@ff.uc.pt

[7] CNC-UC—Center for Neuroscience and Cell Biology, University Coimbra, 3004-517 Coimbra, Portugal

[8] CIBB—Centre for Innovative Biomedicine and Biotechnology, University Coimbra, 3004-548 Coimbra, Portugal

[9] Comprehensive Health Research Center (CHRC), University of Évora, 7004-516 Évora, Portugal; tiago.lima@uevora.pt

[10] Department of Medical and Health Sciences, School of Health and Human Development, University of Évora, 7004-516 Évora, Portugal

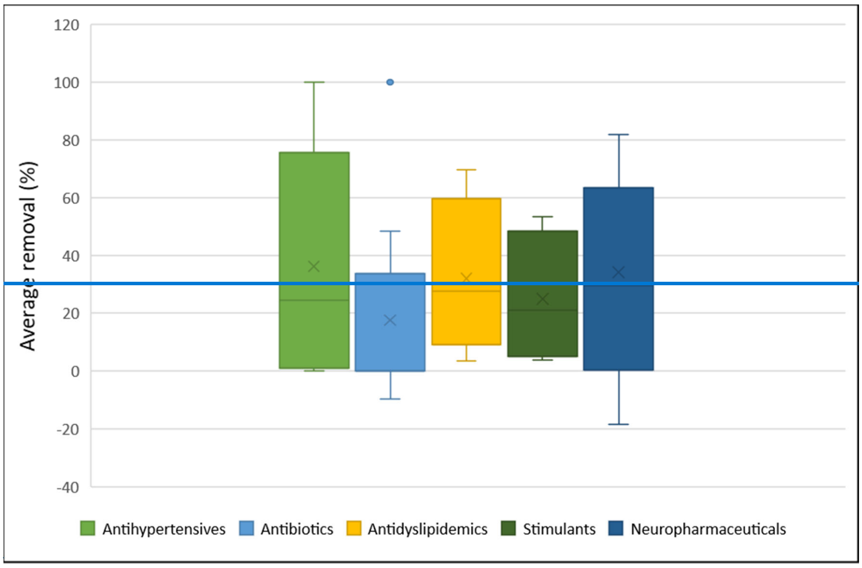

Field Code Changed

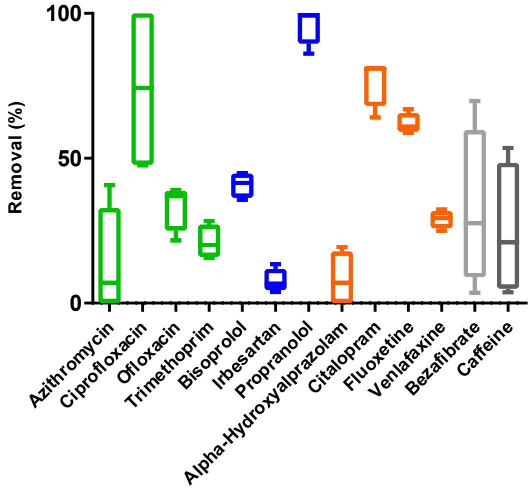

~~(x – average; · – outlier)~~

Figure S1. Average removal for each pharmaceutical.~~Boxplot (minimum, first quartile, median, third quartile and maximum) of removal efficiency for each therapeutic group~~.

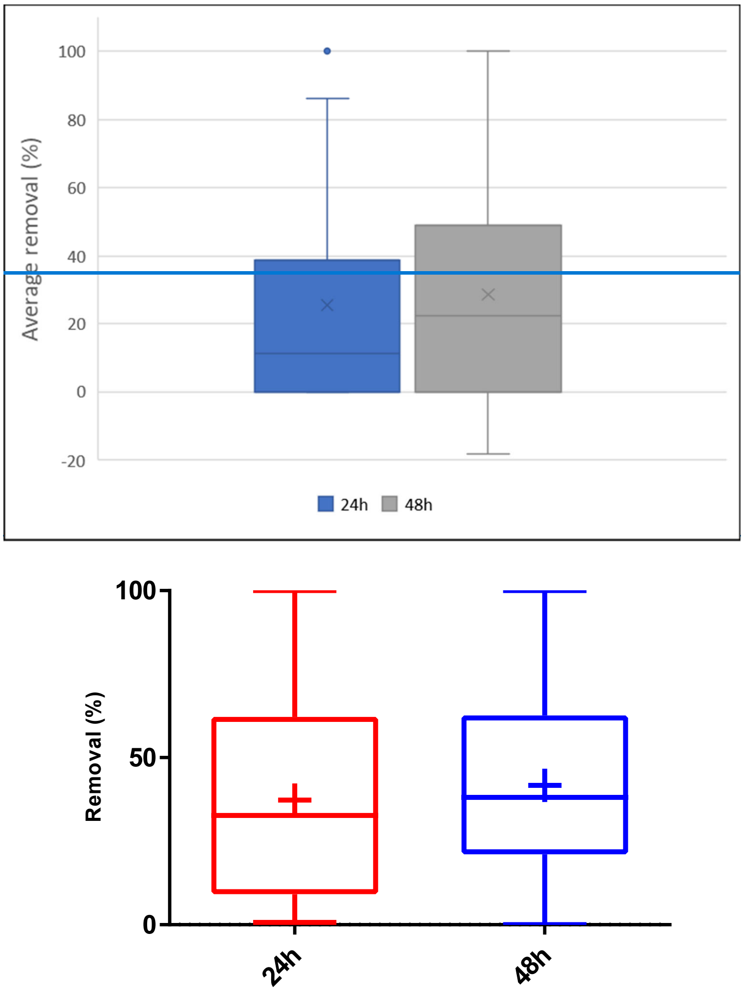

(x – average; · – outlier)

Figure S2. Boxplot showing clam removal efficiency, calculated by comparison with the blank, for each hydraulic retention time. Whiskers represent the minimum and maximum values, boxes indicate the first quartile, median, and third quartile, and the "+" symbol indicates the mean.Boxplot (minimum, first quartile, median, third quartile and maximum) of removal efficiency for each hydraulic retention time.

Field Code Changed

Formatted: English (United Kingdom)

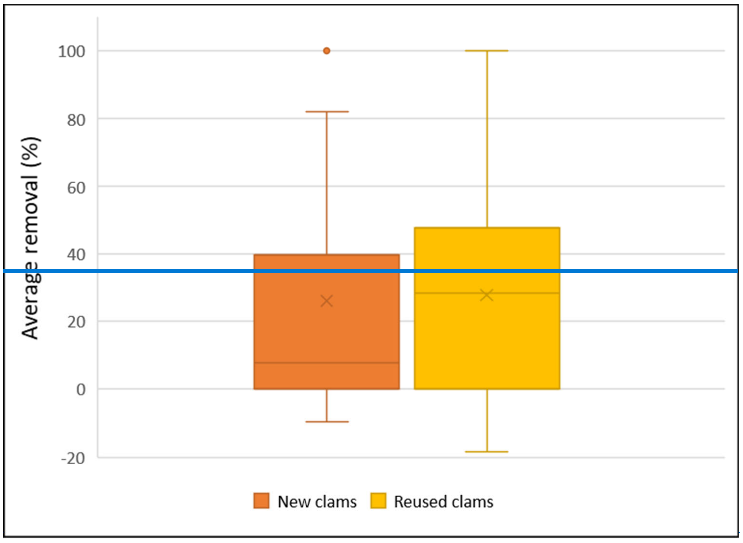

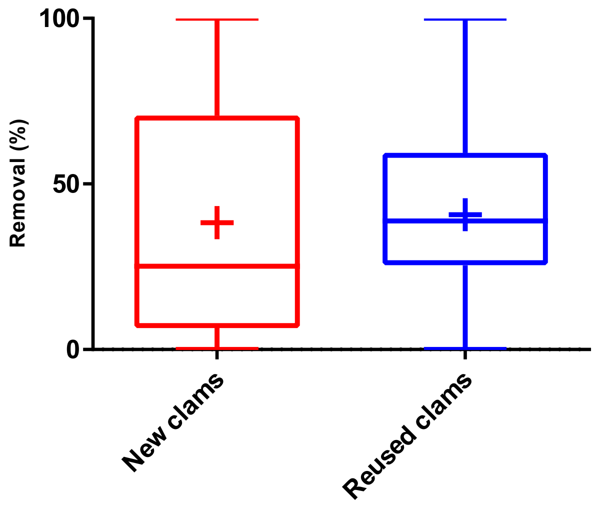

Field Code Changed

~~(x – average; · – outlier)~~

Figure S3. Boxplot showing clam removal efficiency, calculated by comparison with the blank, for new and reused clams. Whiskers represent the minimum and maximum values, boxes indicate the first quartile, median, and third quartile, and the "+" symbol indicates the mean.~~Boxplot (minimum, first quartile, median, third quartile and maximum) of removal efficiency for new and reused clams.~~

Table S1. Pharmaceutical compounds included in the scope of the method, mass detection parameters, and limits of detection (LOD) for each compound in water.

Compound	Molecular Formula	Molecular Weight	$[M+H]^+$	LoD (ng/L)	RT (min)
**Analgesic**					
Acet**aminophen**	$C_8H_9NO_2$	151.0633	152.0706	1.89	2.21
**Antibiotics**					
Amoxicillin	$C_{16}H_{19}N_3O_5S$	365.1045	366.1118	0.11	6.91
**Azithromycin**	$C_{38}H7_2N_2O_{12}$	748.5085	749.5158	0.01	5.04
Benzylpenicillin	$C_{16}H_{18}N_2O_4S$	334.0987	335.1060	0.75	4.49
Ceftiofur	$C_{19}H_{17}N_5O_7S_3$	523.0290	524.0363	0.03	5.85
Cephalexin	$C_{16}H_{17}N_3O_4S$	347.0939	348.1013	0.04	4.85
Chlortetracycline	$C_{22}H_{23}ClN_2O_8$	478.1142	479.1216	1.73	4.78
Cinoxacin	$C_{12}H_{10}N_2O_5$	262.0589	263.0663	0.03	5.25
Ciprofloxacin	$C_{17}H_{18}FN_3O_3$	331.1332	332.1405	3.47	4.47
Danofloxacin	$C_{19}H_{20}FN_3O_3$	357.1488	358.1562	3.25	4.59
Doxycyclin	$C_{22}H_{24}N_2O_8$	444.1532	445.1605	0.28	5.23
Enoxacin	$C_{15}H_{17}FN_4O_3$	320.1284	321.1358	3.33	4.33
Enrofloxacin	$C_{19}H_{22}FN_3O_3$	359.1645	360.1718	2.27	4.66
epi-Chlortetracycline	$C_{22}H_{23}ClN_2O_8$	478.1142	479.1216	1.00	4.54
epi-Tetracycline	$C_{22}H_{24}N_2O_8$	444.1532	445.1605	0.40	4.27
Flumequine	$C_{14}H_{12}FNO_3$	261.0801	262.0874	0.01	6.18
Marbofloxacin	$C_{17}H_{19}FN_4O_4$	362.1390	363.1463	2.51	4.29
Nalidixic acid	$C_{12}H_{12}N_2O_3$	232.0847	233.0921	0.92	6.07
Norfloxacin	$C_{16}H_{18}FN_3O_3$	319.1332	320.1405	1.81	4.41
Ofloxacin	$C_{18}H_{20}FN_3O_4$	361.1437	362.1511	0.99	4.43
Oxolinic acid	$C_{13}H_{11}NO_5$	261.0637	262.0710	3.08	5.50
Oxytetracycline	$C_{22}H_{24}N_2O_9$	460.1481	461.1555	0.24	4.40
Spiramycin	$C_{43}H_{74}N_2O_{14}$	842.5140	843.5213	0.01	5.02
Sulfachloropyridazine	$C_{10}H_9ClN_4O_2S$	284.0134	285.0208	0.43	4.95
Sulfadiazine	$C_{10}H_{10}N_4O_2S$	250.0524	251.0597	0.01	3.81
Sulfadimethoxine	$C_{12}H_{14}N_4O_4S$	310.0735	311.0809	0.30	5.70
Sulfadimidin	$C_{12}H_{14}N_4O_2S$	278.0837	279.091	0.14	4.51
Sulfadoxine	$C_{12}H_{14}N_4O_4S$	310.0735	311.0809	0.03	4.94
Sulfamethizole	$C_9H_{10}N_4O_2S_2$	270.0245	271.0318	0.32	4.52
Sulfamethoxazole	$C_{10}H_{11}N_3O_3S$	253.0521	254.0594	0.03	5.12
Sulfapyridine	$C_{11}H_{11}N_3O_2S$	249.0572	250.0645	0.46	3.80
Sulfaquinoxaline	$C_{14}H_{12}N_4O_2S$	300.0681	301.0754	0.03	5.71
Sulfathiazole	$C_9H_9N_3O_2S_2$	255.0136	256.0209	0.56	3.65
Sulfisomidine	$C_{12}H_{14}N_4O_2S$	278.0837	279.091	0.10	3.42
Sulfisoxazole	$C_{11}H_{13}N_3O_3S$	267.0677	268.075	0.02	5.04
Tetracycline	$C_{22}H_{24}N_2O_8$	444.1532	445.1605	0.48	4.57
Tilmicosin	$C_{46}H_{80}N_2O_{13}$	868.5660	869.5733	0.01	5.40
Trimethoprim	$C_{14}H_{18}N_4O_3$	290.1378	291.1452	0.80	4.24
Tylosin A	$C_{46}H_{77}NO_{17}$	915.5191	916.5264	0.36	5.90
**Anticonvulsants**					
Carbamazepine	$C_{15}H_{12}N_2O$	237.1022	237.1022	0.01	6.08

Gabapentin	$C_9H_{17}NO_2$	172.1332	172.1332	0.81	3.61
Topiramate	$C_{12}H_{21}NO_8S$	340.1061	340.1061	0.03	5.82
**Antidepressants**					
Alpha-Hydroxyalprazolam	$C_{17}H_{13}ClN_4O$	324.0777	325.0851	0.02	6.12
Fluoxetine	$C_{17}H_{18}F_3NO$	309.1340	310.1413	0.01	4.88
Lorazepam	$C_{15}H_{10}Cl_2N_2O_2$	320.0119	321.0192	2.98	4.53
Sertraline	$C_{17}H_{17}Cl_2N$	305.0738	306.0811	0.03	6.13
Venlafaxine	$C_{17}H_{27}NO_2$	277.2041	278.2115	0.02	5.24
**Antihypertensives**					
Furosemide	$C_{12}H_{11}ClN_2O_5S$	331.015	331.015	0.62	6.04
Indapamide	$C_{16}H_{16}ClN_3O_3S$	366.0674	366.0674	0.03	6.27
Irbesartan	$C_{25}H_{28}N_6O$	429.2397	429.2397	0.03	6.31
Losartan	$C_{22}H_{23}ClN_6O$	423.1695	423.1695	0.05	5.77
β-Blockers					
**Atenolol**	$C_{14}H_{22}N_2O_3$	266.1630	267.1703	0.01	3.47
**Bisoprolol**	$C_{18}H_{31}NO_4$	325.2253	326.2326	0.14	5.29
Carvedilol	$C_{24}H_{26}N_2O_4$	406.1892	407.1965	0.83	5.87
Propranolol	$C_{16}H_{21}NO_2$	259.1572	260.1645	0.06	4.39
**Lipid regulators**					
Atorvastatin	$C_{33}H_{35}FN_2O_5$	559.2603	559.2603	8.92	8.46
**Bezafibrate**	$C_{19}H_{20}ClNO_4$	362.1154	362.1154	0.07	6.91
Fenofibrate	$C_{20}H_{21}ClO_4$	361.1201	361.1201	0.02	9.41
Gemfibrozil	$C_{15}H_{22}O_3$	251.1642	251.1642	0.21	4.99
Simvastatin	$C_{25}H_{38}O_5$	419.2792	419.2792	2.80	6.20
**Anti-inflamatory**					
Diclofenac	$C_{14}H_{11}Cl_2NO_2$	295.0166	296.024	0.02	7.70
Ibuprofen	$C_{13}H_{18}O_2$	206.1306	207.138	2.72	6.22
Nimesulide	$C_{13}H_{12}N_2O_5S$	308.0466	309.054	0.03	7.23

Table S2. Mobile phase gradient used during chromatographic analysis.

Time (min)	Mobile phase A: 0.1% formic acid in water (%)	Mobile phase B: acetonitrile (%)
0	97	3
5	40	60
9	0	100
10	97	3
12	97	3

Formatted Table
